# Improving the Quadrupole to Ion Mobility Region in a Digital Quadrupole/Ion Mobility/Orbitrap Mass Spectrometer

**DOI:** 10.1021/jasms.5c00142

**Published:** 2025-10-02

**Authors:** Robert L. Schrader, Gordon A. Anderson, Kacie A. Evans, David H. Russell

**Affiliations:** † Department of Chemistry, 14736 Texas A&M University, College Station, Texas 77843, United States; ‡ GAA Custom Electronics, Kennewick, Washington 99338, United States

**Keywords:** native mass spectrometry, Fourier transform ion mobility, Orbitrap mass spectrometry, quadrupole mass spectrometry, digital mass filter

## Abstract

A quadrupole/drift tube ion mobility/Orbitrap instrument requires pumping from atmosphere to 10^–5^ Torr in the quadrupole analyzer region, back to 1 Torr for the drift tube, and a return to 10^–5^ Torr for the Orbitrap high vacuum region. The Orbitrap high vacuum region contains the transfer multipole, C-trap, and HCD cell. Insufficient pumping between the drift tube and quadrupole leads to helium in the quadrupole analyzer chamber which compromises performance. Additional vacuum regions were added between the drift tube and the analyzer chamber to maintain the analyzer pressure below 5.5 × 10^–5^ Torr with the drift tube pressurized to 1.5 Torr of He. In this configuration, the isolation of a single charge state of C-reactive protein (*m*/*z* 5,000) was demonstrated. Fourier transform ion mobility spectra were acquired with the quadrupole in full scan mode and in the isolation mode. Ion optic voltages were optimized for both helium and nitrogen bath gases in the drift tube so that minimal ion heating was observed entering the drift tube. These instrument modifications enable improved ion transfer efficiency, allowing for better ion isolation by the digital quadrupole ion mobility separation of large protein complexes, as illustrated by the 23+ and 24+ charge states of C-reactive protein.

## Graphical abstract

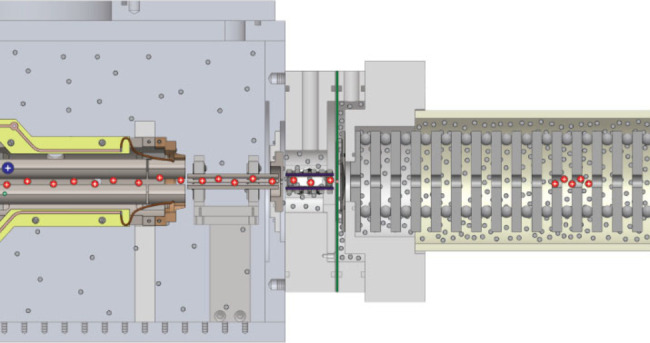

## Introduction

The integration of native mass spectrometry and structural biology has catalyzed improvements in mass spectrometry instrumentation, specifically the development of instruments with increased high *m*/z range and resolving power.^1–9^ Here we describe a series of modifications designed to optimize the digital quadrupole (DigiQ) system for native MS for DT-FT-IMS-EMR Orbitrap MS. Integration of the DigiQ mass analyzer has expanded the capabilities for native MS studies, both in terms of the areas of investigation and the sizes of molecules/complexes that can be studied. The demonstrated capabilities for DigiQ mass selection of a single charge of the 801 kDa GroEL tetradecamer complex facilitates integrating tandem MS^10–14^ and ion mobility spectrometry^15^ studies of large protein complexes. Here we describe recent progress for implementing DigiQ mass selection with drift-tube Fourier-Transform Ion Mobility Spectrometry with high-resolution Orbitrap MS. A periodic focusing drift tube (PF-DT) ion mobility^16–19^ and quadrupole/ion mobility^20^ Orbitrap instrument has been developed for the study of large proteins and protein complexes. The drift tube yields first-principles rotationally averaged cross sections. The Orbitrap has *m*/*z* dependent resolving power, but remains high enough at elevated *m*/*z* (up to 80,000) to support native MS application, including studies of ligand binding with large protein complexes.^2^

The first challenge in quadrupole/drift tube ion mobility instrumentation is the voltage requirements for the drift tube, which increases the requirements of the DC level of the quadrupole before it. Typical field strengths of the drift tube are between 5–15 V cm^–1^ Torr^–1^ which, depending on the length, can be multiple kV.^21,22^ As the mobility resolution scales with the square root of the length, operation at high voltages is desirable. The typical sinusoidally driven quadrupole mass filter scans *m*/*z* by scanning the RF amplitude and the DC voltage between the rod pairs at a fixed RF frequency.^23^ For example, isolation of *m*/*z* 5,000 with sinusoidal quadrupole with an inscribed radius (r_0_) of 5.25 mm operated at 500 kHz would require an RF amplitude of 4976 V_p‑p_ and 835 V between the rod pairs. The voltages necessary to operate the quadrupole are increased to a DC level above that of the entrance of the drift tube. Combined, these voltage requirements can quickly become problematic due to Paschen breakdown. The mass range of the sinusoidally driven quadrupole can be extended by decreasing the drive frequency.^1,24,25^ Alternatively, the digital mass filter operates by scanning the drive frequency at a fixed amplitude which allows for operation at far lower RF amplitudes.^26,27^ The isolation of charge states protein complexes up to *m*/*z* 17,000 with an RF amplitude of 150 V by a digital mass filter has been demonstrated.^28^

A second challenge in the quadrupole/drift tube ion mobility configuration is collisional activation of ions entering the ion mobility region from the quadrupole analyzer region. The ions exit the high vacuum of the quadrupole (ca. 10^–5^ Torr) to form an ion mobility cell in the 1 Torr regime. These disparate pressures are maintained by multiple stages of differential pumping in which separately pumped vacuum regions of the mass spectrometer are separated only by a small aperture between the two regions. These challenges have been shown for quadrupole/traveling wave ion mobility instruments (Waters Synapt).^29–31^ A helium cell before the traveling wave ion mobility cell aids in gently moving ions into the traveling wave ion mobility without causing ion heating.^32,33^ While this is sometimes desirable, i.e. collision induced unfolding,^34^ it is necessary to gently transmit the ions to maintain native-like structures when that is preferred. The transfer optics must be carefully designed to ensure that minimal ion heating occurs on entering the ion mobility device. In developing the DigiQ-IMS system, our design priority was to preserve native or native-like protein conformations by minimizing collisional heating during transfer, even at the expense of achieving maximum sensitivity.

In this work, modifications to a quadrupole/PF-DT IM-Orbitrap instrument are described that improve the vacuum regions between the quadrupole region and the drift tube, which decreased the pressure in the quadrupole analyzer region, ensuring optimum digital quadrupole selection and gentle ion transfer as the pressure increases from ca. 10^–5^ Torr to ca. 1 Torr. Previously, only low resolution mass filtering with ion mobility separation was demonstrated with C-reactive protein, a 115 kDa pentamer complex.^35^ Here, CRP was used to demonstrate the selection of single charge states by a digital quadrupole combined with ion mobility.

## Experimental Section

### Methods

Human recombinant C-reactive protein (CRP, 1 mg in 140 mM NaCl, 20 mM Tris-HCl, and 2 mM CaCl_2_, at a pH of 7.5), ammonium acetate, and LC grade water were purchased from Millipore-Sigma (Burlington, MA). The solution was buffer exchanged using Micro Biospin P-6Gel Columns (Bio-Rad, Hercules, CA) into 200 mM ammonium acetate. The protein concentration was adjusted to 4 μM. Glass capillaries (Sutter Instrument, Navajo, CA, 1.5 mm o.d., 0.86 mm i.d., 10 cm length) were pulled using a Sutter Instrument P-1000 micropipette puller. The solution was loaded into pulled glass capillaries and high voltage (1.1–1.5 kV) applied through platinum wire (0.3 mm, Thermo Scientific).

### Instrumentation

Ions enter through a heated capillary (120 °C) into an RF ion funnel (472 kHz, 250 V_p‑p_) maintained at a pressure of 1.1 Torr. The heated capillary is offset from the instrument axis by 0.375 in. to remove the line-of-sight from the subsequent vacuum regions. The ion funnel is followed by a 3.5 mm r_0_ square quadrupole in a vacuum region maintained at 0.3 Torr by an additional mechanical pump. The following three vacuum regions are pumped by a three-inlet TMH 261–250–010 P3P (Pfeiffer, Asslar, Germany) turbomolecular pump. The first turbopump region is maintained at 1.1 × 10^–3^ Torr and contains a Thermo Fisher 4 mm r_0_ segmented quadrupole mass filter (q1, Part Number 80100–60109, 1.37 MHz, 2500 V_p‑p_) and a 2.75 mm r_0_ octopole ion guide (MP1, 784 kHz, 450 V_p‑p_). The second turbopump region is maintained at 4.8 × 10^–5^ Torr and contains a Thermo Fisher 5.25 mm r_0_ segmented quadrupole mass filter (Q2, Part Number 80133–60100, 100 V_0‑p_ rectangular waveform of variable duty cycle) and a 2.75 mm r_0_ octopole ion guide (MP2, 786 kHz, 450 V_p‑p_). In addition to the triple stage turbopump, the Q2/MP2 region is also pumped by a second 200 L/s turbomolecular pump. The rectangular waveforms were generated by an Astraea waveform generator (GAA Custom Electronics, Kennewick, WA).^36^ The following region is pumped by the interstage of the turbomolecular pump to an estimated pressure of 10^–3^ Torr and contains a 3.5 mm r_0_ segmented planar quadrupole ion guide (MP3, 526 kHz, 400 V_p‑p_), modified from a previously described design,^37^ followed by the drift tube entrance (800 μm) pumped by a mechanical pump to approximately 8 × 10^–2^ Torr.^38^ The 55 cm periodic focusing drift tube is pressurized to approximately 1.135 Torr of helium or nitrogen, measured by a capacitance manometer located at the rear of the drift tube. The drift gas was added at the front of the drift tube. Following an 800 μm skimmer aperture, the ions enter a 4.75 mm r_0_ octopole ion guide (902 kHz, 250 V_p‑p_) to exit the HCD cell of a Thermo Fisher Exactive Plus Orbitrap EMR (Bremen, Germany). All pressure measurements (except the quadrupole and drift tube) were made by Pirani gauges. The quadrupole analyzer region pressure was measured by a cold cathode gauge. A full diagram of the instrument is shown in Figure S1. All RF and DC voltages were generated by Modular Intelligent Power Supplies (MIPS) systems and High Q RF heads (GAA Custom Electronics, Kennewick, WA).

A Siglent Technologies SDG1032X (Shenzhen, China) arbitrary waveform generator was used to generate the 5–5005 Hz frequency sweep over 8 min for FT-IM where the TTL signal from the waveform generator was used to trigger a FET pulser (GAA Custom Electronics) to gate ions synchronously at the entrance and exit of the drift tube.

### Data Processing

Quadrupole mass spectra were acquired by iterating through a range of quadrupole drive frequencies and acquiring the Orbitrap mass spectra at each frequency step. The acquisition was automated through the Thermo Fisher Xcalibur and MIPS software by using an Xcalibur sequence file and a contact closure signal generated by the MIPS. Following acquisition, ‘.RAW’ files for each quadrupole frequency were converted to mzXML with MSConvert^38^ and analyzed using MATLAB (The Mathworks, Inc., Natick, MA) with the Bioinformatics toolbox. Ion mobility data files were processed as previously described using custom Python scripts.^17–20^

## Results and Discussion

### Instrument Modifications

The primary challenge in a quadrupole/ion mobility/Orbitrap configuration (opposed to ion mobility/quadrupole/Orbitrap) is the disparate pumping requirements of the quadrupole mass filter and the ion mobility device, i.e., a drift tube. As the quadrupole is generally operated in the 10^–5^–10^–6^ Torr regime, and the drift tube is generally operated in the 1 Torr regime, it becomes necessary to span pressures from 1 Torr to 10^–5^ Torr from the ion source to the quadrupole, return to 1 Torr for the drift tube, and then return to 10^–5^ Torr for the Orbitrap high vacuum chamber. The Orbitrap high vacuum chamber contains the transfer multipole, the C-trap, and the HCD cell.

In a previous instrument configuration,^20^ the drift tube was directly interfaced with the Q2/MP2 region. As the drift tube was pressurized with helium, a large increase in the pressure of the Q2/MP2 region from helium leaking to the quadrupole analyzer region was observed. To mitigate this, three modifications were made to the instrument shown in Figure 1a: (1) a second turbomolecular pump was added to the quadrupole analyzer region; (2) the drift tube was modified to add rough pumping in the drift tube entrance region (this region was not previously directly pumped); and (3) a new vacuum region with a quadrupole ion guide (MP3) was added between the Q2/MP2 region and the drift tube entrance, each separated by 2 mm apertures. The q1/MP1 and Q2/MP2 regions are pumped by a triple-stage turbomolecular pump, where the first interstage is a KF-25 flange, which now pumps the MP3 region. Two sets of voltage parameters were used: one optimized to minimize potential differences between regions and another designed to induce activation in the MP3 region by increasing all prior voltages, creating a larger voltage drop into MP3. These parameters, shown in Figure 1b, were evaluated with the drift tube filled with either helium or nitrogen. With these modifications, two differentially pumped vacuum regions separate the Q2/MP2 region from the drift tube. The drift tube entrance and Q2/MP2 pressures were measured as a function of the drift tube pressure up to 1.5 Torr (Figure 1c). With the drift tube pressurized to 1.5 Torr of helium, the Q2/MP2 pressure only reaches 5.0 × 10^–5^ Torr, which is suitable for operation of the quadrupole mass filter.

**Figure 1:**
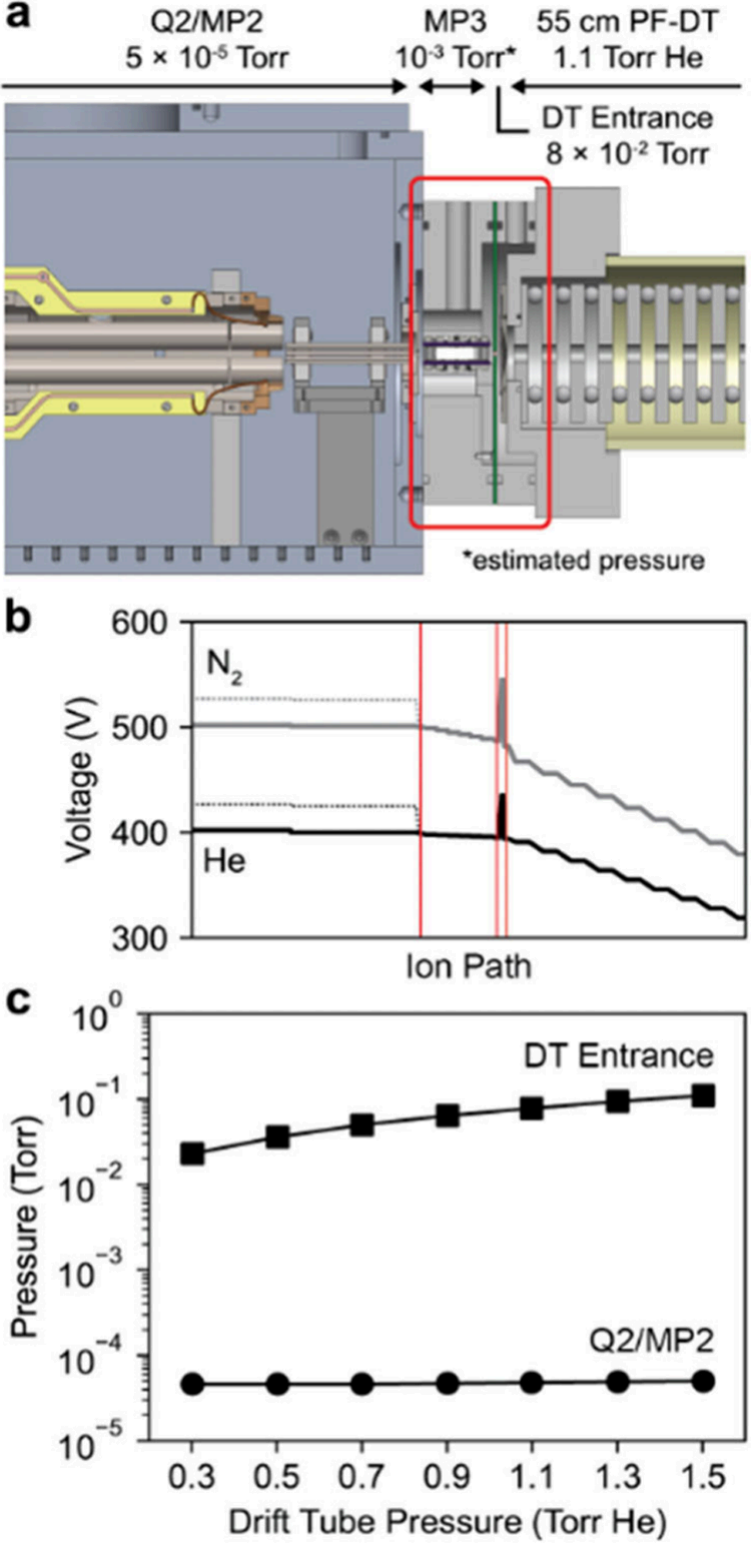
(a) Schematic showing operating pressures after DT addition with additional pumping and the new region MP3. (b) Plot of corresponding operating voltages in line with the schematic, with dashed lines indicating activating voltages, when the DT is filled with either helium or nitrogen. The voltage spike seen at the DT entrance is indicative of the gating electrode for FT-IM. (c) Plot of pressures in the DT entrance and Q2/MP2 regions as the pressure in the DT is varied.

### Ion Mobility Spectra in Helium

To acquire a full scan mass spectrum in the Orbitrap, a 300 kHz, 100 V_0‑p_, 50.0/50.0 duty cycle waveform was applied to the digital quadrupole (Figure 2a). In this case, ions of *q* value <0.712 are stable which corresponds to a low mass cut off of *m*/*z* 533. The full scan mass spectrum is shown in Figure 2a. This allows for the determination of collision cross sections of each charge state of CRP. The drift tube field strength was 5.63 V cm^–1^ Torr^–1^ with a helium bath gas. The collision cross section for the 23+ and 24+ charge state was determined to be 63.5 nm^2^ and 64.1 nm^2^, respectively (Figure 2b). This is consistent with previously published data for this complex^20,39^ and demonstrates that the instrument can gently transmit the ions into the drift tube without unfolding or fragmentation.

**Figure 2:**
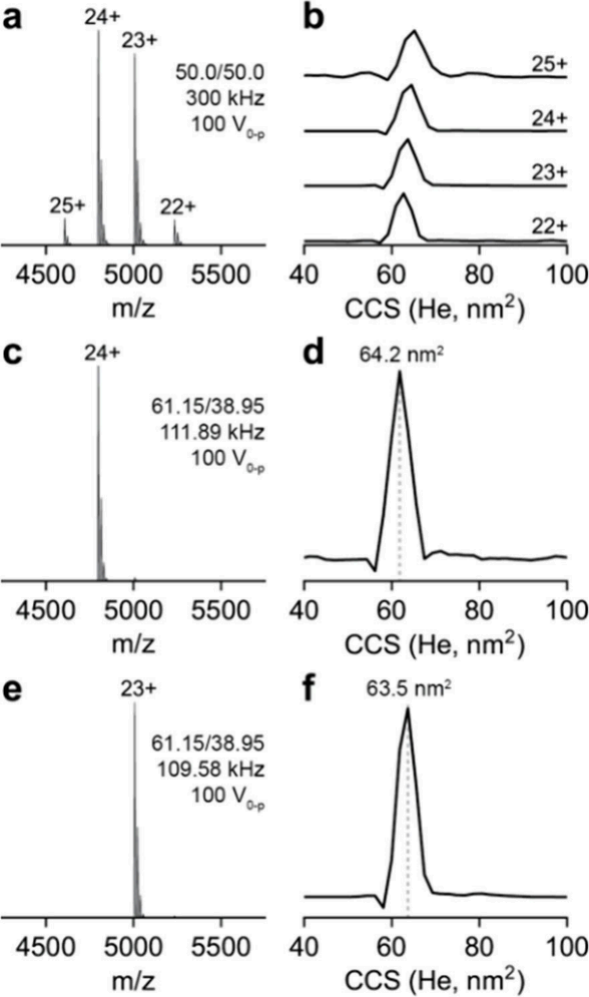
(a) Orbitrap mass spectrum and (b) collision cross sections for each charge state, 22–25+, in the mass spectrum of CRP with helium bath gas in the drift tube. (c) Orbitrap mass spectrum after isolation of the 24+ charge state using the digital mass filter (Q2) and (d) collision cross section of the 24+ charge state. (e) Orbitrap mass spectrum after isolation of the 23+ charge state using the digital mass filter (Q2) and (f) collision cross section of the 23+ charge state.

**Figure 3:**
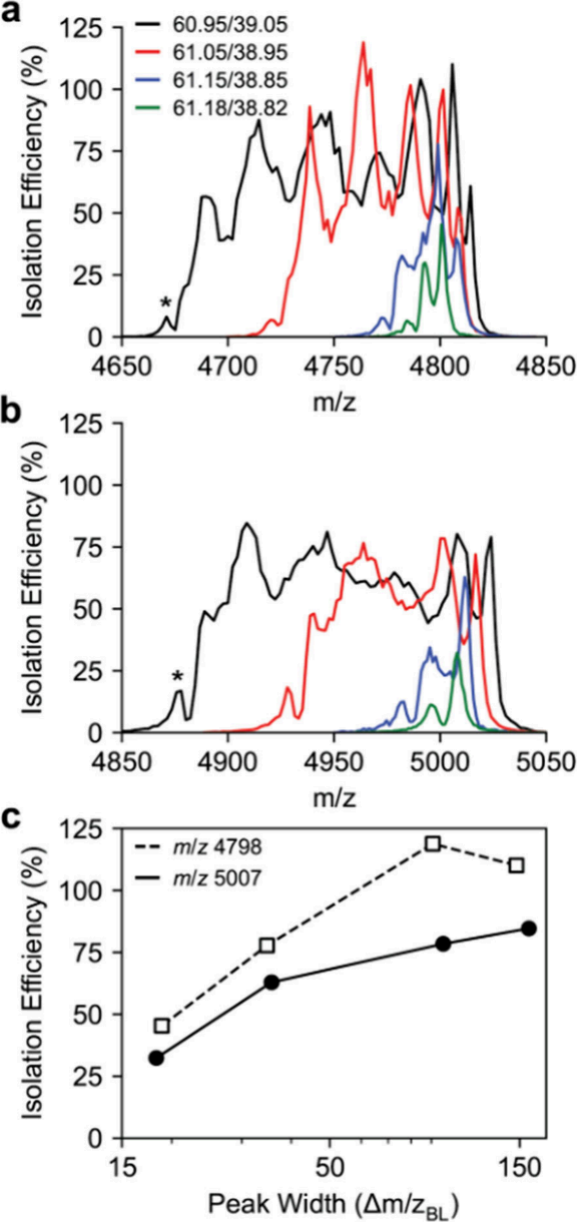
Digital mass filter spectra for the (a) 24+ (*m*/*z* 4798) and (b) 23+ (*m*/*z* 5007) charge state of CRP corresponding to four duty cycles with increasing resolving power. (c) Peak width versus isolation efficiency for the 24+ and 23+ charge state of CRP. The isolation efficiency is defined as the difference in the maximum Orbitrap intensity for the isolated ion versus the Orbitrap intensity at a 50.0/50.0 duty cycle and 300 kHz.

To isolate the 24+ charge state (*m*/*z* 4898), a 111.98 kHz, 100 V_0‑p_, 61.15/38.85 duty cycle waveform was applied to the digital quadrupole while all other parameters remained constant (Figure 2c). The collision cross section determined at 64.2 nm^2^ (Figure 2d) matches the cross section determined from the full scan data. Alternatively, the 23+ charge state (*m*/*z* 5007) was isolated by applying a 109.58 kHz, 100 V_0‑p_, 61.15/38.85 duty cycle waveform while all other parameters remained constant (Figure 2e). The determined collision cross section of 63.5 nm^2^ (Figure 2f) matches the cross section determined from the full scan data.

Unlike the commercial Orbitrap ion source, mass spectra obtained through the ion mobility have a large fraction of the CRP ions bound to a 371 Da adduct species. This is attributed to the “cold” ion source used with the ion mobility instrument, in which the short (∼5 cm) transfer path within the ion funnel reduces the time ions spend in this high-pressure region, thereby minimizing collisions and associated collisional heating.^16^

### Digital Quadrupole Peak Shapes

To determine experimental digital quadrupole resolving power, the duty cycle was set to 60.95/38.95, 61.05/38.95, 61.15/38.85, or 61.18/38.82, which corresponds to theoretical *q*/Δ*q* baseline resolving powers (RP_BL_) of 32.83, 53.46, 143.03, and 286.69, respectively. The quadrupole drive frequency was scanned and an Orbitrap mass spectrum taken at each frequency. The quadrupole peak shape was determined from the extracted Orbitrap intensity of the ion of interest at each quadrupole drive frequency, which was converted to *m*/*z*. Quadrupole peak shapes were measured for the 24+ (Figure 3a) and 23+ (Figure 3b) charge states of CRP, corresponding to *m*/*z* 4798 and *m*/*z* 5007, respectively. The isolation efficiency as a function of peak width is shown in Figure 3c. The isolation efficiency was determined from the difference in Orbitrap intensity in the isolated spectrum versus the Orbitrap intensity at a 50.0/50.0 duty cycle at 300 kHz. As this compares the signal intensity at two different drive frequencies and therefore *q* values, differences in the transmission make the isolation efficiencies less representative of the quadrupole performance since the quadrupole acceptance area depends on the drive frequency. This definition was chosen as it represents the difference in Orbitrap intensity for typical operating parameters. As the quadrupole peak shapes are collected using an XCalibur method, the collection of the entire peak shape takes approximately 30 min. Any changes in the intensity of the nanoelectrospray source will be reflected in the isolation efficiency and can result in isolation efficiencies greater than 100%.

An ideal quadrupole peak shape is rectangular, as the signal would be zero when the ion is outside the stable *q* window, have a maximum value within the stable *q* window, and return to zero signal when outside the stable *q* window. The experimentally observed peak shapes have two nonidealities: (1) the ion signal oscillates within the stable window, referred to as peak splitting or peak structure, and (2) an extra peak outside of the stable window, notated with an asterisk in Figure 3a and b, is observed on the low *m*/*z* side of the peak. Despite the nonidealities, the experimentally determined resolving powers match expected values calculated from the stability diagram. Peak splitting has been observed previously in the first stability zone,^40^ higher stability zones,^41,42^ and with digital quadrupoles in the first stability zone.^43^ In this case, the peak splitting is a result of the 800 μm entrance aperture of the drift tube. The small aperture at the drift tube entrance is necessary to maintain appropriate pressure in the quadrupole analyzer region (∼5 × 10^–5^ Torr) and to maintain a static pressure in the drift tube (∼1–1.5 Torr). Splitting results from variance in ion beam width as the *q* value changes within the stable window– this leads to variation in the numbers of ions entering the drift tube with ion *q* value. This makes operation of the instrument difficult as it can be challenging to find the optimal quadrupole drive frequency to achieve maximum signal intensity. As the quadrupole is only operated at 100 V_0‑p_, the postfilter is ineffective at refocusing the ion beam to its original diameter.^43^ Increasing the RF voltage on the quadrupole, and consequently the postfilter, would reduce this effect but comes at the expense of power draw of the waveform generator.

An extra peak outside of the stable window on the low *m*/*z* side is observed at each *m*/*z* and resolving power. This can be attributed to both the ion kinetic energy and low *y* dimension ion secular frequencies. Low *m*/*z* ions are being lost in the *y*-dimension near the β_*y*_ = 0 boundary. Given the drive frequency of approximately 110 kHz, the secular frequencies in this dimension are near zero, and some of these ions will be transmitted outside of a stable *q* value because of the low number of RF cycles in the quadrupole. This can be mitigated by decreasing the ion translational energy to increase the number of RF cycles or increasing the RF voltage which would consequently increase the drive frequency and secular frequencies. As with the peak splitting, this comes at the expense of power draw of the waveform generator.

### Comparison of Operation in Nitrogen and Helium

Nitrogen is a popular choice as an ion mobility bath gas due to its low cost and higher breakdown voltage when compared to helium and is often used in commercially available ion mobility systems. As the drift tube is maintained at a much higher pressure than the vacuum regions around it, the background gas of the surrounding vacuum regions is composed primarily of drift gas. Furthermore, ion velocities are greatly reduced in nitrogen compared to helium due to the difference in their size. Therefore, the differences between ion optic voltages entering the drift tube (from the Q2/MP2 Exit to the drift tube) were increased to maintain ion transmission. A comparison on optimized voltages injection into the drift tube in both helium and nitrogen bath gas is shown in Figure 1b. The primary concern when exiting the quadrupole region to the increasing pressure regions in the drift tube is the potential for ion activation if the ion velocity is too high. The collision cross section distribution of the 24+ charge state of CRP at the optimized transmission voltages is shown in Figure 4a, which is consistent with a folded conformation. Increasing the voltage drop in the MP3 region by 50 V (Figure 4b) does not yield a significant change in the collision cross section. This is attributed to the fact the helium does not impart a significant amount of energy into an ion the size of CRP for fragmentation or collisional cooling. Corresponding mass spectra, shown in Figure S2, show a minimal change in the number of adducted species. It was observed that these ions were not thermalized again until they had entered the drift tube. In the nitrogen bath gas at voltages optimized for transmission (Figure 4c), the collision cross section is again consistent with the folded conformation. Unlike helium, increasing the voltage drop in the MP3 region by 50 V (Figure 4d) yields an unfolded conformation due to collisional activation. This is attributed to the increased energy imparted by collisions with nitrogen gas. Unlike that for helium, the reduced adduct intensity is observed in the Orbitrap mass spectra with this activation (Figure S2).

**Figure 4:**
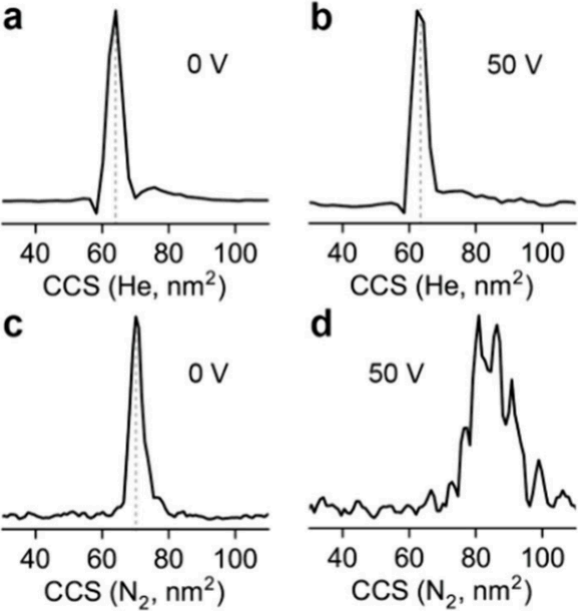
Collision cross section in helium for the 24+ charge state of CRP with (a) 0 V and (b) 50 V of activation in MP3. No change in the collision cross section was observed with this activation. Alternatively, the collision cross section in nitrogen for the 24+ charge state of CRP with (c) 0 V and (d) 50 V of activation in MP3. Nitrogen drift gas also fills MP3 with nitrogen, which yields more activating collisions as the ions enter the elevated pressure regions after the digital mass filter.

To monitor ion heating within the drift tube, the field strength was varied in both the helium and nitrogen bath gas. The intensity of the 371 Da adduct species varies with the amount of collisional activation. In helium bath gas, the Orbitrap mass spectrum (Figure 5a) and arrival time distribution (Figure 5b) at 3 V cm^–1^ Torr^–1^ and the Orbitrap mass spectrum (Figure 5c) and arrival time distribution (Figure 5d) at 7 V cm^–1^ Torr^–1^ (Figure 5c) show that there is a significant reduction in the adduct distribution at higher ion mobility field strengths. As all other interoptic voltages remained constant, this activation was attributed solely to the drift tube. In nitrogen bath gas, the Orbitrap mass spectrum (Figure 5e) and arrival time distribution (Figure 5f) at 7 V cm^–1^ Torr^–1^ and the Orbitrap mass spectrum (Figure 5g) and arrival time distribution (Figure 5h) at 12 V cm^–1^ Torr^–1^ (Figure 5c) show the same decrease in the adduct distribution at higher field strength. Interestingly, the number of adducted species at 7 V cm^–1^ Torr^–1^ in nitrogen is greater than the number of adducted species at 3 V cm^–1^ Torr^–1^ in the helium bath gas. Unlike entering the drift tube where nitrogen was determined to be harsher on the ions, within the drift tube helium yields greater activation as has been previously observed.^44^ Within the periodic focusing drift tube ions experience alternating focusing and defocusing fields which can contribute to ion activation.^45^ In nitrogen bath gas, the ions are more efficiently cooled to the center of the drift tube where they experience lower fields than ions in helium.

**Figure 5:**
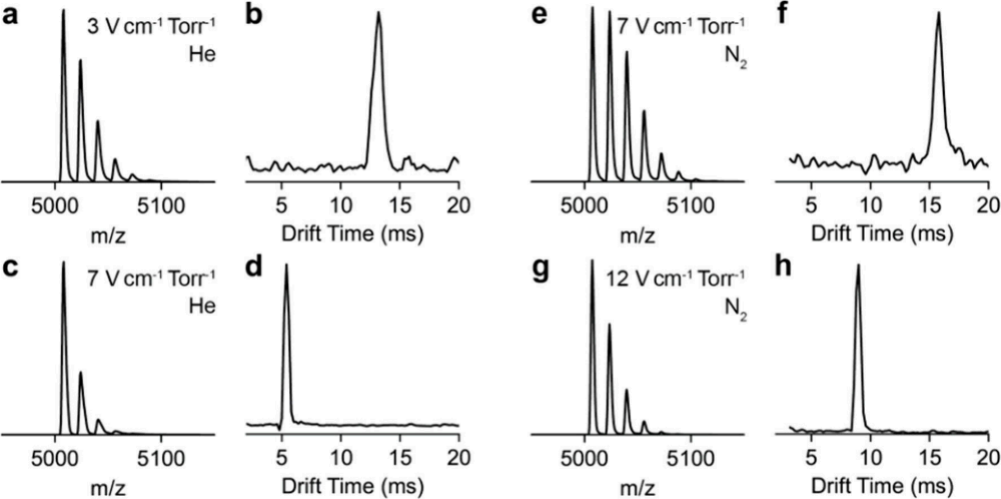
(a) Orbitrap mass spectrum for the 23+ charge state of CRP where the drift strength was 3 V cm^–1^ Torr^–1^ in helium bath gas and (b) the corresponding arrival time distribution. (c) Orbitrap mass spectrum of the 23+ charge state of CRP where the drift strength was 7 V cm^–1^ Torr^–1^ in helium bath gas and (d) the corresponding arrival time distribution. (e) Orbitrap mass spectrum for the 23+ charge state of CRP where the drift strength was 7 V cm^–1^ Torr^–1^ in nitrogen bath gas and (f) the corresponding arrival time distribution. (g) Orbitrap mass spectrum of the 23+ charge state of CRP where the drift strength was 12 V cm^–1^ Torr^–1^ in nitrogen bath gas and (h) the corresponding arrival time distribution.

### Ion Mobility Spectra in Nitrogen

While a 5.63 V cm^–1^ Torr^–1^ drift strength was used in helium bath gas, the drift strength was increased to 6.84 V cm^–1^ Torr^–1^ while maintaining the pressure in the drift tube at 1.135 Torr. Full scan Orbitrap mass spectra with nitrogen bath gas in the drift tube are shown in Figure 6a. The collision cross sections for CRP in nitrogen bath gas were determined for the 24+ and 23+ charge state to be 70.4 nm^2^ and 69.5 nm^2^, respectively, (Figure 6b) which are consistent with previously published values.^39^

**Figure 6:**
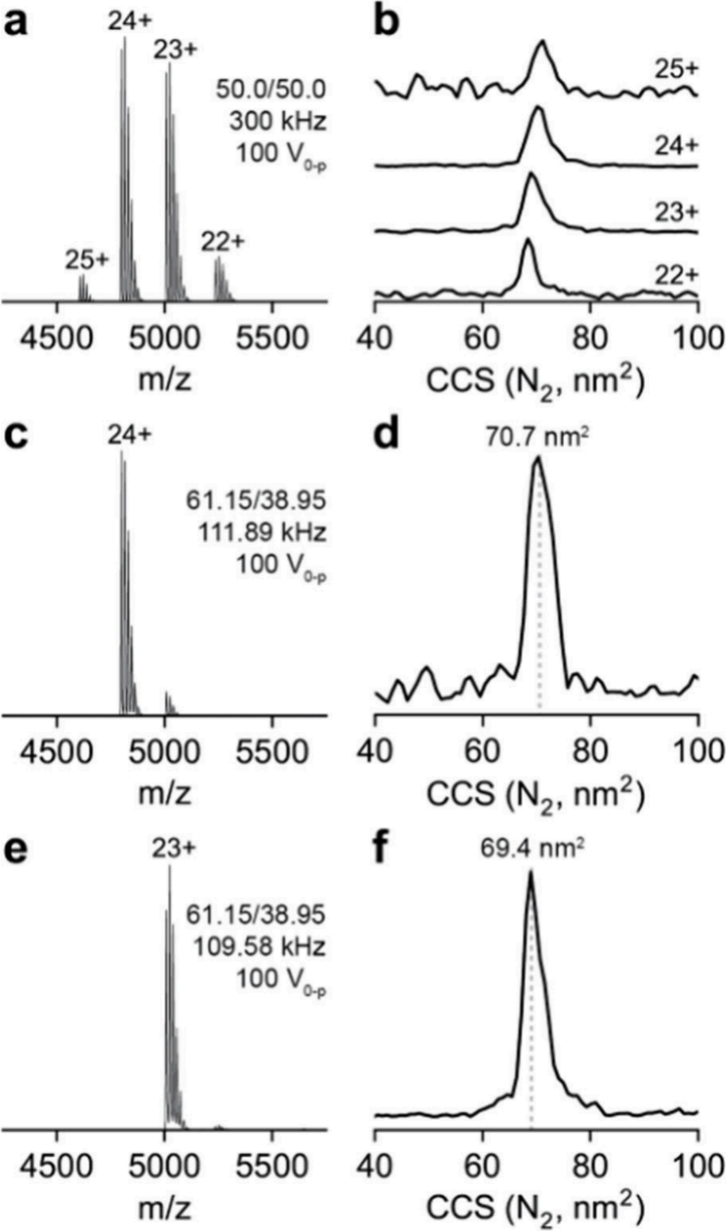
(a) Orbitrap mass spectrum and (b) collision cross sections for each charge state, 22–25+, in the mass spectrum of CRP with nitrogen bath gas in the drift tube. (c) Orbitrap mass spectrum after isolation of the 24+ charge state using the digital mass filter (Q2) and (d) collision cross section of the 24+ charge state. (e) Orbitrap mass spectrum after isolation of the 23+ charge state using the digital mass filter (Q2) and (f) collision cross section of the 23+ charge state.

Using the same parameters as with helium bath gas, the 24+ (Figure 6c) and 23+ (Figure 6e) charge state of CRP was selected using the digital mass filter. The cross section for the selected 24+ charge state was 70.7 nm^2^ (Figure 6d) and the selected 23+ charge state was 69.4 nm^2^ (Figure 6f) which match the cross sections obtained from the full scan. While the Orbitrap mass spectra look very similar, an increase in the abundance of leakage (transmission of 23+ when 24+ is isolated) is observed in the nitrogen bath gas. This observation is attributed to the increase in collisional cooling in MP3 due to the change in gas composition. As the ions are dispersed in space in the quadrupole, ions that are formally unstable can be transmitted, provided they can exit the mass filter prior to hitting the rod pairs. These ions will have large radial dimensions that must be focused to less than 800 μm prior to entering the drift tube to be transmitted through the drift tube. As the MP3 gas is primarily gas leaking from the drift tube, the change to nitrogen bath gas is expected to increase the collisional cooling in this multipole. This suggests that the nitrogen bath gas may better reflect the distribution of ions leaving the quadrupole.

## Conclusions

The quadrupole/ion mobility/Orbitrap configuration is a challenge due to the disparate pumping requirements of the quadrupole (10^–5^ Torr) and the drift tube (1 Torr). Previous iterations of a home-built quadrupole/ion mobility/Orbitrap instrument suffered from high pressures in the quadrupole analyzer region due to leakage of the drift gas into this region. Modifications to the instrument to add additional differential pumping between the quadrupole and the drift tube decreased the pressure in the quadrupole analyzer region to achieve improved digital quadrupole isolation performance.

The digital quadrupole mass filter can readily isolate individual charge states of a 115 kDa protein complex, CRP, and perform ion mobility. Quadrupole peak shapes were determined by scanning the digital quadrupole drive frequency which showed previously observed splitting due to the small apertures required to maintain the required pressures.

The ion mobility drift tube can be operated with either helium or nitrogen bath gas and was able to transmit native-like conformations in either case. Nitrogen bath gas is much more readily able to collisionally activate ions entering the drift tube but is gentler for ions within the drift tube. Helium behaves oppositely and is unable to collisionally activate ions entering the drift tube but is much harsher on ions within the drift tube.

Looking ahead, the current DigiQ-IMS configuration meets our present research needs, but we anticipate making targeted modifications as new analytical challenges arise. One potential avenue for improvement is increasing the ion mobility resolution by implementing a longer drift tube; however, the voltage requirements of this upgrade in combination with the DigiQ currently exceed our power supply and hardware capabilities. As future needs emerge, we will adapt and refine the platform accordingly.

## Supplementary Material



## Notes

Full instrument diagram; mass spectra for varying levels of MP3 activation (PDF)

## §

Thermo Fisher Scientific, San Jose, California 95134, United States
