# Supplementary material for: Improving the Quadrupole to Ion Mobility Region in a Digital Quadrupole/Ion Mobility/Orbitrap Mass Spectrometer
Source: J Am Soc Mass Spectrom. 2025 Oct 2;36(11):2389–96. doi: 10.1021/jasms.5c00142 (PMC12598848; doi:10.1021/jasms.5c00142)
Supplement: Supplementary file 1 [file js5c00142_si_001.pdf]

Supplementary Information

Improving the Quadrupole to Ion Mobility Region in a Digital Quadrupole/Ion Mobility/Orbitrap Mass Spectrometer

Robert L. Schrader[1‡], Gordon A. Anderson[2], Kacie A. Evans[1], and David H. Russell[1*]

[1]Department of Chemistry, Texas A&M University, College Station, TX 77843
[2]GAA Custom Electronics, Kennewick, WA 99338
[‡]Current address: Thermo Fisher Scientific, San Jose, CA, US 95134

*Corresponding author; email: russell@chem.tamu.edu

Table of Contents

	Page
Figure S1	S2
Figure S2	S3

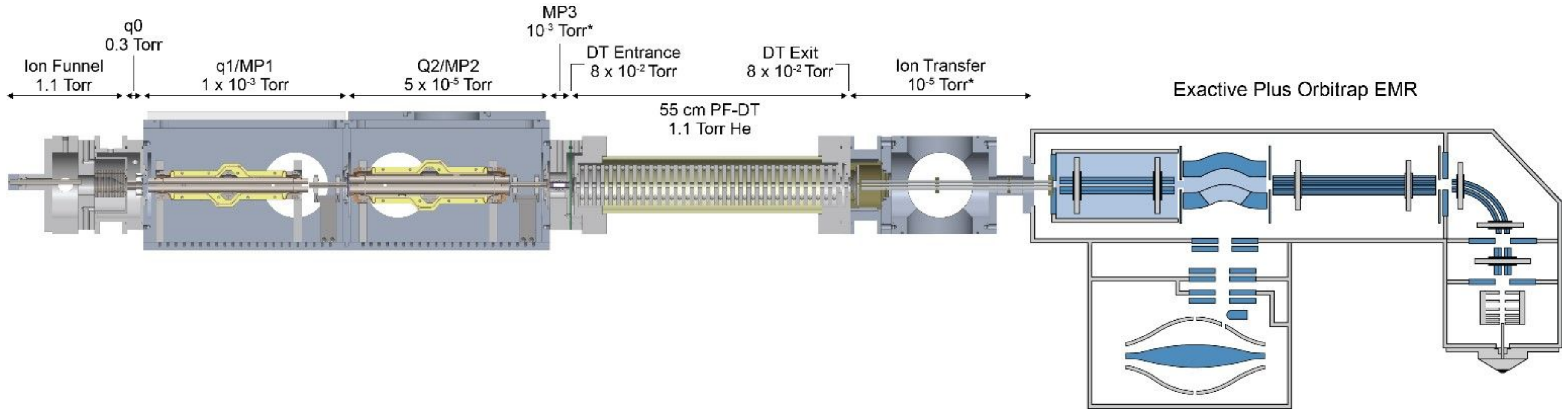

Figure S1. Solidworks rendering of the instrument interfaced with the rear of the HCD cell of the Exactive Plus Orbitrap EMR with major components labeled with gauge pressures of each vacuum region. Note that pressures labeled with * are estimated.

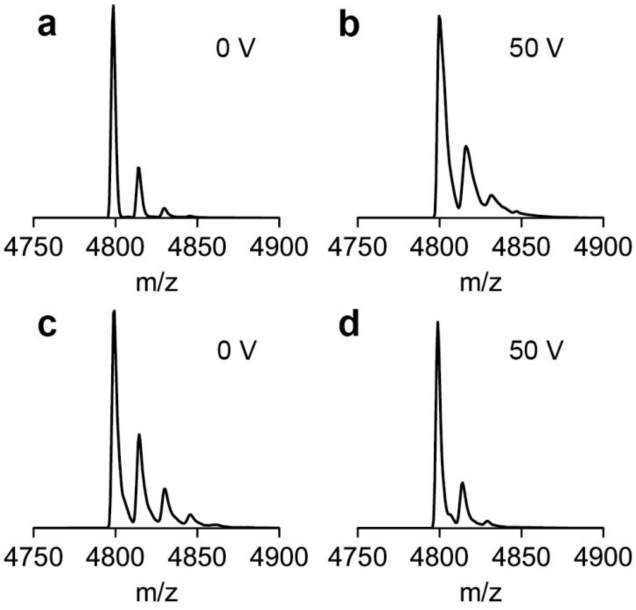

Figure S2. Orbitrap mass spectra for the 24+ charge state of CRP with helium in the drift tube with (a) 0 V and (b) 50 V of activation in MP3 and Orbitrap mass spectra for the 24+ charge state of CRP and with nitrogen in the drift tube with (c) 0 V and (d) 50 V of activation in MP3.
